# An Observational Study to Assess the Reflection of Vipassana Meditation (VM) on the Perception of "Mindfulness" as a Tool for Health Promotion Among Beneficiaries of VM

**DOI:** 10.7759/cureus.89865

**Published:** 2025-08-12

**Authors:** Tilottama Wankhade, Sujata Lavangare, Akanksha P Dani

**Affiliations:** 1 Public Health, Taluka Health Office, Dharni, IND; 2 Community Medicine, Seth Gordhandas Sunderdas Medical College and King Edward Memorial Hospital, Mumbai, IND; 3 Community Medicine, All India Institute of Medical Sciences, Nagpur, Nagpur, IND

**Keywords:** acceptance, awareness, mental health, mindfulness, philadelphia mindfulness scale, therapy psychology, vipassana meditation

## Abstract

Introduction

Vipassana meditation (VM) is an ancient meditation technique based on the teachings of Gautama Buddha that involves becoming aware of the present moment and the associated experiences. Therapeutic psychology practices such as mindfulness show promise for improving mental health in terms of stress reduction, emotional regulation, and overall well-being. Despite the increasing use of mindfulness-based interventions, relatively little is known about the unique contribution of VM to the development of mindfulness.

Objectives

The objective of this study was to measure the effect of VM on two key components of mindfulness, awareness and acceptance, using the Philadelphia Mindfulness Scale (PHLMS).

Materials and methods

A pre-experimental (pre-test and post-test design) study was conducted at Khadavali Vipassana Centre in Thane, India, over a period of 18 months. The sample (N=156) included participants from diverse socio-demographic backgrounds who engaged in VM for 10 days. The participants were assessed using the PHLMS, a self-report instrument for measuring mindfulness as present-centered awareness and non-judgmental acceptance. Both descriptive and inferential statistics and Wilcoxon’s signed-rank test were used to analyze the pre- and post-test mindfulness scores.

Results

A significant increase in mindfulness was observed post-intervention across all participant groups. Specifically, the average total score for mindfulness increased from 61.47 to 81.09 (p < 0.001). Both the awareness subscale and the acceptance subscale increased significantly from the pre-intervention stage to the post-intervention stage, awareness from 30.76 to 41.11 and acceptance from 30.70 to 39.99. A significant proportion of the participants progressed from a lower level of mindfulness to a higher level, with 74.3% achieving a high level of mindfulness after the VM retreat.

Conclusion

VM appears to promote mindfulness, mainly because of the effect of acceptance. This study contributes to the literature by demonstrating the value of VM as an intervention in therapeutic work for enhancing mindfulness and, thereby, mental health.

## Introduction

Vipassana meditation (VM) is one of the oldest forms of meditation practiced in India. It was discovered and taught by Gautama Buddha some 2,500 years ago [1]. “Vipassana” is a meditation or Dhamma term that is derived from two words: “vi” refers to the three aspects of mentality and physicality (impermanence, suffering, and the absence of self or ego), and “passana” refers to correct insight or realization achieved through intense focus and awareness of mental and bodily processes. Thus, Vipassana involves the correct comprehension of the three mental and physical aspects [2].

In the Pali language, the term describes the “insight” to see things as they really are. Vipassana, also known as “insight awareness” or “mindfulness meditation,” is, then, a scientific technique for investigating natural rules (known as Dhamma) within the context of an individual’s mind and body [3].

Mindfulness, on the other hand, involves paying attention to the present moment in a non-judgmental manner and is also receiving recognition for contributing to mental health and well-being [4]. This therapeutic psychology practice is employed especially for stress reduction, emotional control, and mental well-being. As a form of meditation, it is characterized by deep tranquility, awareness, and insight and has the potential to alter perspectives about the mind.

Recent literature indicates that mindfulness-based interventions are efficacious for addressing a wide array of conditions across various demographics [5,6]. These conditions include clinical illnesses and symptoms such as anxiety [7-9], the relapse of depression [10,11], depressive symptoms [12], and stress [13-15]. Mindfulness interventions have also achieved favorable medical and well-being outcomes such as reducing chronic pain [16], improving quality of life [10,17], and relieving psychological or emotional discomfort [18,19]. VM exerts these therapeutic effects by increasing mindfulness.

The tools used to measure mindfulness include the Mindful Attention Awareness Scale, the Five-Facet Mindfulness Questionnaire (FFMQ), the Cognitive and Affective Mindfulness Scale-Revised, the Freiburg Mindfulness Inventory, the Langer Mindfulness Scale, the Solloway Mindfulness Survey, the Kentucky Inventory of Mindfulness Skills, the Automatic Thoughts Questionnaire, the Philadelphia Mindfulness Scale (PHLMS), the State Mindfulness Scale, and the Toronto Mindfulness Scale. The PHLMS is a valid instrument that has been widely used in research to assess mindfulness, in particular, the two core dimensions of present-centered awareness (PCA) and acceptance, which encapsulate its foundational aspects. PCA is the ability to attend to the present moment, and acceptance is the capacity to become aware of thoughts, emotions, and physical sensations without judgment or becoming attached to them.

In this study, we used the PHLMS to explore the effect of VM, which deepens connections to the present and fosters nonjudgmental attention, on these two aspects of mindfulness [20]. We conducted the study because, despite the growing body of evidence demonstrating the benefits of mindfulness, there remains a dearth of research on the impact of specific meditation practices, including Vipassana, on mindfulness and individuals’ perceptions. Our aim was to understand the benefits of specific and disciplined VM practice, as it is currently taught, for the development of mindfulness. The findings may inform meditation-based therapies for promoting mental wellness and changing individuals’ perceptions and may increase the understanding of the influence of VM on mindfulness and changes in cognition. The empirical evidence and experiential information that this study provides thus stand to contribute to the empirical and experiential literature on meditation and the evidence base for further mindfulness research and interventions. Our objective was to assess changes in the level of mindfulness in individuals as a result of VM.

## Materials and methods

Regarding the study design, we conducted a pre-experimental study involving an intervention using a pre-test and a post-test. There was no control group. The location for the study was the Khadavali Vipassana Centre in the city of Thane in the state of Maharashtra in India. The participants were drawn from various backgrounds and attended meditation practice at the center. 

The study began after the Institutional Ethics Committee approved it and lasted for 18 months. Data was collected before the start of the VM session and after the completion of 10 days of the VM session. Because of the dearth of literature about VM, we calculated the sample size based on the availability of the participants and improvement in mindfulness reported by authorized persons from the VM center. Specifically, we calculated the sample size as

$n \geq \left[ z_{1a/2}(\Psi H) + z_{1-\beta}\sqrt{(\Psi H)^2(\Psi 1)^2 \div (\Psi 1)^2 \div \text{discord} / (\Psi 1)^2 \div \text{discord}} \right]$

where the z-values account for confidence and power, and the parameters ΨH, ΨΞ, and discord serve to adjust for variance, effect sizes, and measurement inconsistencies. By inserting the findings of pre-test mindfulness = 45% and post-test mindfulness = 55% into the equation, we determined the appropriate sample size to be 156. The sample size was calculated using OpenEpi software using the above formula.

Our sampling technique was complete enumeration. Thus, we considered for inclusion in the study all of those who fulfilled the inclusion criteria, attended 10 full days of VM sessions, and consented to participate. Each VM session included 50 participants. We interviewed 10 participants from each group who fulfilled the inclusion criteria until the sample size was reached in order to reduce social desirability bias. The inclusion criteria were participants of Indian origin who had not previously attended a VM session and could read or understand Hindi, Marathi, and/or English.

The study tool for the first part of the study was interviews with the participants to collect their general and socio-demographic information. For the second part of the study, we used self-assessment based on the pre-validated PHLSM to measure mindfulness in terms of acceptance and awareness. We translated the scale from English into Hindi and Marathi and got it validated by the experts. The PHLSM consists of two sets of 10 questions. The total score of the scale is 100, with 50 points each for acceptance and awareness. The scale was validated in terms of internal consistency, with Cronbach’s alpha values ranging from 0.75 to 0.86 for awareness and 0.75 to 0.91 for acceptance [21].

We determined the effectiveness of the VM sessions on the basis of increases in the number of participants at a “high level of mindfulness.” The operational grading of mindfulness levels was as follows: (i) a PHLMS score of 49 or less was classified as a low level of mindfulness, (ii) a PHLMS score of 50 to 74 was classified as a medium level of mindfulness, and (iii) a PHLMS score of 75 or greater was classified as a high level of mindfulness.

Our operational definitions were as follows. “Vipassana” is, as mentioned, a Pali word meaning literally “inward vision” that, in this context, indicates seeing things as they really are. “Mindfulness” is the psychological process of nurturing and paying attention to experiences occurring in the present moment, or “living in the present,” which can be developed through the practice of meditation and other training. “Awareness” is “the continuous monitoring of ongoing internal and external stimuli” [2]. “Acceptance” is “a non-judgmental stance toward one’s experience” [4].

Intervention given to the participants

Interventions given to the participants include a routine schedule for 10 days from 4:00 am to 9:30 pm (Table 1).

**Table 1:** Schedule of participants attending vipassana meditation (VM) for 10 days

Timing	Activity
4:00 a.m	Morning wake-up bell
4:30 — 6:30 a.m.	Meditate in the hall or in your room
6:30 — 8:00 a.m	Breakfast break
8:00 — 9:00 a.m.	Group meditation in the hall
9:00 — 11:00 a.m.	Meditate in the hall or in your room according to the teacher’s instructions
11:00 —12 noon	Lunch break
12:00 —1:00 p.m.	Rest, and interviews with the teacher
1:00 — 2:30 p.m.	Meditate in the hall or in your room
2:30 — 3:30 p.m.	Group meditation in the hall
3:30 — 5:00 p.m.	Meditate in the hall or in your room according to the teacher's instructions
5:00 — 6:00 p.m.	Tea break
6:00 — 7:00 p.m.	Group meditation in the hall
7:00 — 8:15 p.m	Teacher’s discourse in the hall
8:15 — 9:00 p.m	Group meditation in the hall
9:00 — 9:30 p.m.	Question time in the hall
9:30 p.m	Retire to your room; lights out.

Ethical considerations

We obtained permission to conduct the study and ethical approval for it from the Institutional Ethics Committee (IEC(I)/OUT/345/2018). We also obtained permission from the Khadavali Vipassana Centre. Information about the purpose of the study, the procedure, and the risks and benefits was distributed to all of the participants, and informed consent documents were obtained from each of them.

Study procedure

**Figure 1:**
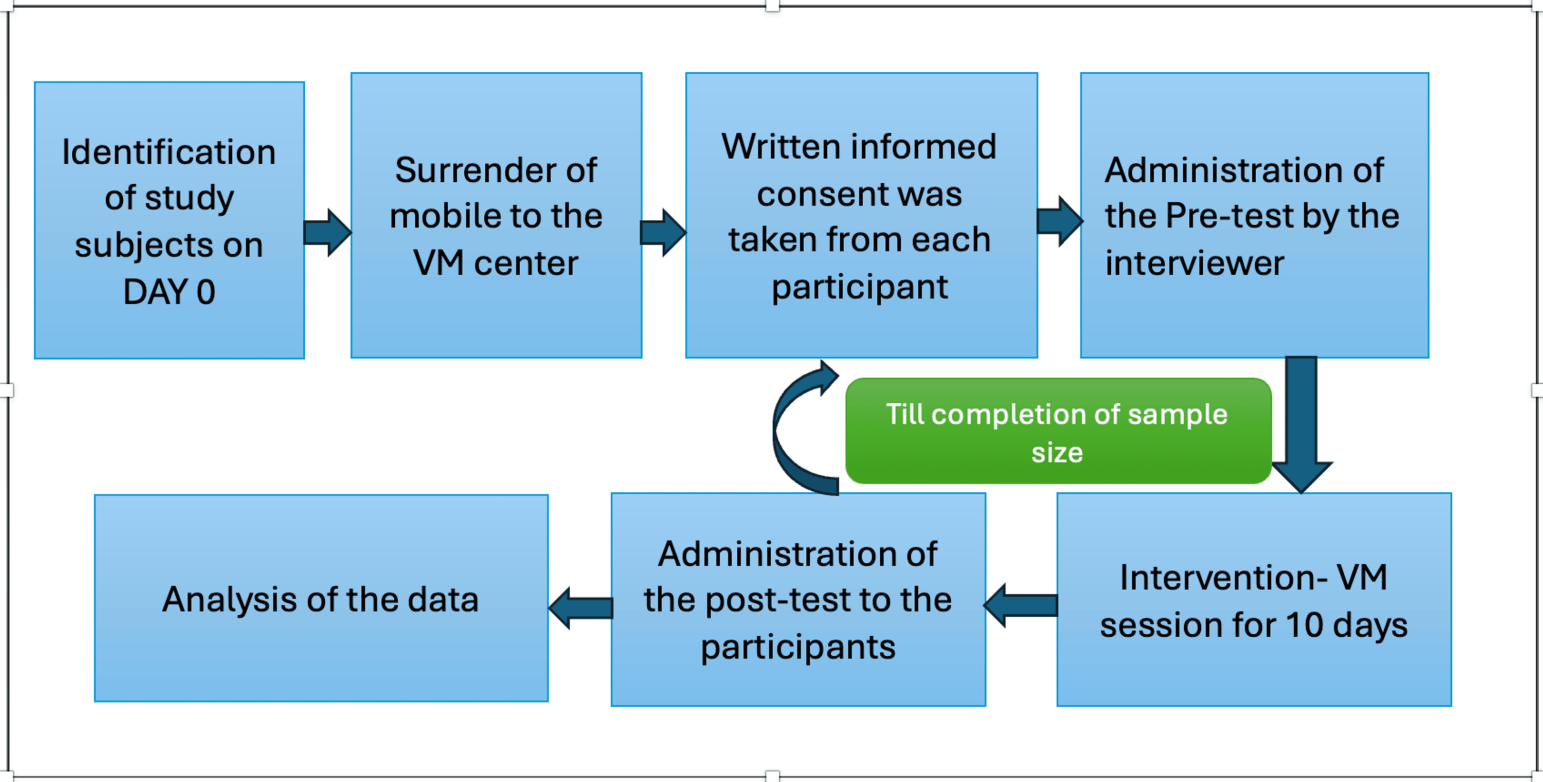
Procedure of the study VM: Vipassana meditation

After joining the Vipassana centre, participants were asked to submit their mobile phones to achieve the complete dedication of participants and to get the maximum benefit of VM. Complete relevant history was taken by the investigator before starting the meditation sessions and after completion of 10 days of sessions (Figure 1).

Data analysis

We analyzed the data using IBM SPSS Statistics for Windows, Version 22 (Released 2013; IBM Corp., Armonk, New York, United States). The descriptive statistics were presented using the mean, median, and range, and the categorical variables were presented in the form of percentages. We analyzed the PHLSM and pre- and post-test scores using the Wilcoxon signed-rank test.

## Results

The study was carried out at the Khadavali Vipassana Centre. There were 156 participants, of whom four (2.56%) could not be followed up for the post-test because they left the VM session before the required 10 days of meditation practice. The following observations are from the pre- and post-test data of the remaining 152 participants.

Analysis of the data indicates that, of 152 study participants, 53.28% (81) were female and 46.71% (71) were male. Overall, nearly half of the participants belonged to the age group of 30-59 years (i.e., 49.3%), followed by the age group of 18-29 years (41.4%). The mean age of the participants in this study was 36.77 years, with a minimum of 19 years and a maximum of 67 years. The range of age was 48 years, and the S.D. was 13.67 years. Most of the participants (77) were married (i.e., 50.7%). The “other” participants in the marital status column included divorced, widow/widower, engaged to be married, and separated. Out of a total of 152, 8.6% (i.e., 7) of the female participants were widows, and one (i.e., 1.4%) male participant was a widower. Apart from this, only one (i.e., 1.2%) female participant was found to be separated. Of the female participants, 6.2% (i.e., 5) were divorced, and only 1.4% (i.e., 1) were engaged. 

In the study, no male participants were found to be divorced or separated. Most of the participants belonged to the Hindu religion (i.e., 57.2%), followed by the Buddhist religion (i.e., 38.2%. The “other” participants in the religion column belonged to the Jain, Muslim, or Christian religions. Around 55.9% (85) were non-working. These non-working participants included homemakers, students, and retired and unemployed individuals. Among the 67 participants who were working, nearly half (i.e., 49%, 33) were working in the private sector, of whom 13 were female and 20 were male. The “self-employed” accounted for 37% (i.e., 25) of the working participants. Only 14% (9) of the participants were working in the government sector, four female participants and five male participants. Among the 85 nonworking participants, most were students (i.e., 52%, 44), 19 female participants, and 25 male participants, followed by female homemakers (i.e., 33%, 28). Regarding socio-economic status, most of the participants (80.9%) belonged to class I of the socioeconomic classification by B. G. Prasad, followed by class II (15.1%) and class III (3.3%). None of the participants belonged to class IV or class V (Table 2).

**Table 2:** Distribution of socio-demographic characteristics of the participants according to gender

	Female (N=81)	Male (N=71)	Total (N=152)
Age (Years)			
18 TO 29	31(38.3%)	32(45.1%)	63 (41.4%)
30 TO 59	41 (50.6%)	34 (47.9%)	75 (49.3%)
60 and Above	9 (11.1%)	5 (7.0%)	14 (9.2%)
Marital Status			
Married	44 (54.3%)	33 (46.5%)	77 (50.7%)
Not married	24 (29.6%)	36 (50.7%)	60 (39.5%)
Other	13 (16.0%)	2 (2.8%)	15 (9.8%)
Religion			
Hindu	42 (51.9%)	45 (63.4%)	87 (57.2%)
Buddhist	35 (43.2%)	23 (32.4%)	58 (38.2%)
Other	4 (4.9%)	3 (4.2%)	7 (4.7%)
Working Status			
Working	27 (33.3%)	40 (56.3%)	67 (44.1%)
Non-working	54 (66.6%)	31 (43.6%)	85 (55.9%)
Socio-economic Status (B G Prasad classification)			
Class I	63 (77.8%)	60 (84.5%)	123 (80.9%)
Class II	15 (18.5%)	8 (11.3%)	23 (15.1%)
Class III	3 (3.7%)	2 (2.8%)	5 (3.3%)

**Figure 2:**
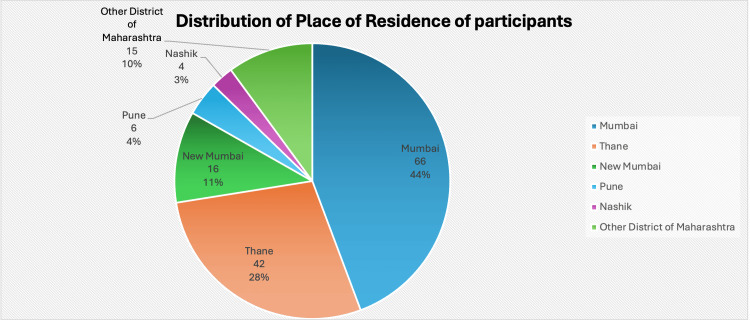
Distribution of place of residence of participants

We observed that a plurality of the participants, 43.4%, were from Mumbai, 27.6% were from Thane, and only 2% were from outside the state of Maharashtra. The participants were from both rural and urban areas (Figure 2).

Pre- and post-intervention results of the PHLSM, with a total score of 100, were obtained. Since the PHLMS is a chart type of scale, the statistical analysis was done using Wilcoxon’s signed-rank test. The scale was pre-validated with Cronbach's alpha values ranging from 0.75 to 0.86 for awareness and 0.75 to 0.91 for acceptance. In the current study, the alpha value for awareness was 0.747 and that for acceptance was 0.743, which was in the acceptable range [20]. Also, Table 3 shows that the total median score after VM increased from 62.00 (IQR 55.5 to 68) to 81.00 (IQR 76 to 87). The mean score of the PHLMS increased from 61.47 to 81.09 (with S.D. 10 and 7.58) before and after the VM sessions, respectively (Table 3).

**Table 3:** Descriptive statistics of pre- and post-intervention of Philadelphia Scale of Mindfulness (PHLSM) scores among participants

	Pre-VM Score				Post-VM score			
	Mean	SD	Median	IQR	Mean	SD	Median	IQR
Overall PLHSM Score (Total Score = 100)	61.47	10.00	62.00	55.5-68	81.09	7.58	81.00	76 - 87
Awareness subscale (Total Score = 50)	30.76	5.64	31.00	27.25-34	41.11	4.26	41.00	38 - 44
Acceptance Subscale (Total Score = 50)	30.70	5.81	31.00	-	39.99	4.06	40.00	-

The findings presented in Table 4 show that most of the participants (149) had a higher total PHLSM score on the post-test than on the pre-test, but two participants had lower post-test scores. Only one participant did not have any change in the total score before and after the VM sessions. The number of participants was significantly higher in the positive ranks than in the negative ranks, and those who had ties, suggesting positive change among participants in terms of mindfulness. The positive ranks were significantly higher in the post-tests overall as well as on both subscales.

**Table 4:** Distribution of Wilcoxon signed rank test and statistics for total PHLSM for group of students, working, non-working and retired participants. *Wilcoxon-signed rank test; a: Based on negative ranks; z: test statistics

Scale		Total Post-test Score - Total Pre-test Score		Ties	Total	Z	P-value*
		Negative Ranks	Positive Ranks				
Overall PHLSM	N	2	149	1	152	- 10.630 ^a^	0.000
	Mean Rank	9.00	76.90				
	Sum of Ranks	18.00	11458.00				
Awareness subscale	N	3	149	0	152	- 10.622 ^a^	0.000
	Mean Rank	14.50	77.75				
	Sum of Ranks	43.50	11584.50				
Acceptance subscale	N	2	149	1	152	- 10.486^ a^	0.000
	Mean Rank	49.00	76.36				
	Sum of Ranks	98.00	11378.00				

Comparison of the data before and after the VM indicated striking improvements in each group of participants. For the student participants, the pre-VM median was 64.50 (IQR 58.25-69.75) while the post-VM median increased to 82.50 (IQR 79-88), with standard deviations of 9.67 and 6.75 before and after the VM, respectively. For the working participants, the pre-VM median was 61 (IQR 52-66) while the post-VM median was 81 (IQR 75-86), with standard deviations of 10.38 and 7.80, respectively. This group also showed a significant increase in the post-VM minimum and maximum scores. For the unemployed participants, the pre-VM median was 63 (IQR = 56.25-69) while the post-VM median was 79 (IQR = 75.25-84.75), with standard deviations of 9.42 and 8.29 before and after the application of the VM, respectively. For the retired participants, the pre-VM median was 60 (IQR = 54.25, 66) while the post-VM median was 80 (IQR = 72.75, 86.75), with standard deviations of 9.142 and 7.25, respectively. Similar statistics were calculated for both subscales (Table 5).

**Table 5:** Distribution of descriptive statistics for pre- and post-VM total score by PHLSM (n=152) VM: Vipassana Meditation; PHLSM: Philadelphia Mindfulness Scale

Scale	Descriptive Statistics	Students (n=44)		Working (n=67)		Non-working (n=29)		Retired (n=12)	
		Pre-VM score	Post-VM score	Pre-VM score	Post-VM score	Pre-VM score	Post-VM score	Pre-VM score	Post-VM score
Overall PLHSM score	Mean	68.41	82.86	59.73	80.45	63.55	80.58	59.00	79.67
	Median	64.50	82.50	61.00	81.00	63.00	79.00	60.00	80.00
	S.D.	9.67	6.75	10.38	7.80	9.42	8.29	9.14	7.25
	IQR	58.25 to 69.75	79.00 to 88.00	52.00 to 66.00	75.00 to 86	56.25 to 69.00	75.00 to 84.75	54.25 to 66.00	72.75 to 86.75
Awareness subscale score	Mean	31.30	42.07	30.12	40.81	31.76	40.55	30.00	40.00
	Median	31.00	42.00	31.00	41.00	30.00	40.00	30.00	40.00
	S.D.	5.49	3.76	5.84	4.51	5.60	4.53	5.19	3.80
	IQR	28.25 to 34.00	39 to 46	26.00 to 34.00	38 to 44	28.00 to 35.00	38.00 to 44.50	27.50 to 32.75	38.00 to 44.00
Acceptance subscale score	Mean	32.11	40.80	29.61	39.64	31.79	39.97	29	39
	Median	32.50	41.00	30	40	32	39	29.50	39.50
	S.D.	5.41	3.78	6.06	4.19	5.85	4.45	4.51	3.69
	IQR	29 to 36	38 to 43	25 to 34	38 to 42	27 to 36.50	37 to 42	24.75 to 33.50	36.25 to 42.00

Table 6 depicts the significant increase in positive ranks in all groups. Only one working participant had the same pre- and post-test scores. Also, there was a statistically significant increase in the level of mindfulness of the participants in each group after the VM interventional session, with p<0.000 (sig. 2-tailed), which is less than the 0.01 level of significance according to the Wilcoxon signed-rank test.

**Table 6:** Distribution of the Wilcoxon signed rank test and statistics for total PHLSM for a group of students, working, non-working and retired participants. a. Total Post-test Score < Total Pre-test Score; b. Total Post-test Score > Total Pre-test Score; c. Total Post-test Score = Total Pre-test Score; d. Based on negative ranks.

		Pre-VM score – Post-VM score					
	Groups	Negative Ranks	Positive Ranks	Ties	Total (n)	Z	p
Total PHLSM	Students	0^a^	44^b^	0^c^	44	-5.781^d^	0.000
	Working	0^a^	67^b^	0^c^	67	-7.118^d^	0.000
	Non-working	0^a^	29^b^	0^c^	29	-4.704^d^	0.000
	Retired	0^a^	12^b^	0^c^	12	-3.062^d^	0.002
Awareness subscale	Students	0^a^	44^b^	0^c^	44	-5.781^d^	0.000
	Working	1^a^	66^b^	0^c^	67	-7.106^d^	0.000
	Non-working	2^a^	27^b^	0^c^	29	-4.501^d^	0.000
	Retired	0^a^	12^b^	0^c^	12	-3.059^d^	0.002
Acceptance subscale	Students	0^a^	44^b^	0^c^	44	-5.786^d^	0.000
	Working	1^a^	66^b^	0^c^	67	-6.948^d^	0.000
	Non-working	1^a^	27^b^	1^c^	29	-4.340^d^	0.000
	Retired	0^a^	12^b^	0^c^	12	-3.072^d^	0.002

**Figure 3:**
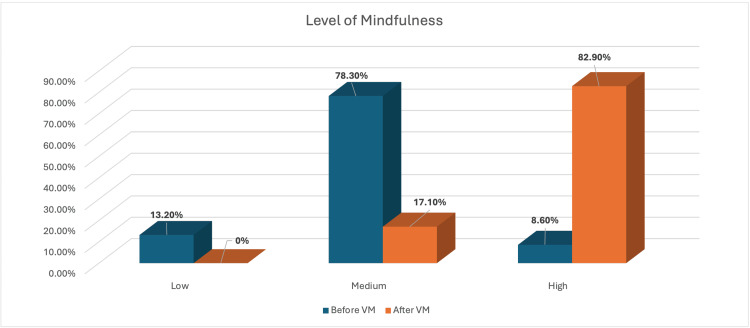
Overall change in the level of mindfulness among participants attending the VM session VM: Vipassana meditation

The proportion of participants with a high level of mindfulness increased by 74.3% after the VM sessions (Figure 3).

Regarding changes in the participants’ levels of mindfulness after the VM sessions, the number of participants with a high level of mindfulness increased by 79.5%, 67.1%, 79.3%, and 83% in the students, employed, non-working, and retired groups, respectively, (Table 7).

**Table 7:** Distribution of study participants according to the level of mindfulness in various groups VM: Vipassana meditation

	Group of participants							
	Students (n=44)		Working (n=67)		Non-working (n=29)		Retired (n=12)	
Level of mindfulness	Pre-VM	Post-VM	Pre-VM	Post-VM	Pre-VM	Post-VM	Pre-VM	Post-VM
Low	2 (6.9%)	00 (00%)	14 (20.9%)	00 (0%)	2 (6.9%)	00 (00%)	14 (20.9%)	00 (0%)
Medium	38 (86.4%)	5 (11.4)	48 (71.6%)	17 (25.4%)	38 (86.4%)	5 (11.4)	48 (71.6%)	17 (25.4%)
High	4 (9.1%)	39 (88.6%)	5 (7.5%)	50 (74.6%)	4 (9.1%)	39 (88.6%)	5 (7.5%)	50 (74.6%)

## Discussion

The aim of this pre-experimental study was to investigate the impact of VM on mindfulness using the PHLMS to assess awareness and acceptance before and after a 10-day VM retreat. The concept of mindfulness, defined as non-judgmental awareness and attention to the present, is increasingly recognized as a therapeutic intervention to enhance emotional regulation, alleviate symptoms of anxiety and depression, and foster overall well-being. The PHLMS measures an individual’s PCA and capacity for nonjudgmental acceptance of thoughts, emotions, and physical sensations [21]. It has been widely used in mindfulness research, so its reliability in evaluating the essential elements of mindfulness attributes is proven.

Research shows that practicing VM is linked to activity in certain parts of the brain, such as the prefrontal and anterior cingulate cortex and thicker areas in the brain that help with attention, and more grey matter in the right insula and hippocampus has been observed among those who meditate regularly. A clinical study of healthy subjects suggested that VM can enhance mature defenses and coping styles, thereby helping individuals deal judiciously with day-to-day stressors [22].

The present study clearly demonstrated that VM significantly improved awareness among the participants, who included students, employed and unemployed individuals, and retired individuals. The mean PHLMS score rose from 61.47 before the VM retreat to 81.09 afterward, indicating a substantial increase in the participants’ mindfulness levels. The awareness and acceptance subscales changed notably, with awareness increasing from a mean of 30.76 before the retreat to 41.11 afterward and acceptance increasing from 30.70 before to 39.99 afterward. These results support the notion that VM helps increase awareness of the present moment and acceptance of thoughts and feelings without judgment [23].

Pradhan et al. measured mindfulness using a short version of the FFMQ (the FFMQ-SF) and the Satisfaction with Life Scale to assess life satisfaction and found higher levels of mindfulness and life satisfaction in the participants who meditated regularly. These scores suggest a direct relationship between mindfulness and life satisfaction, and the strong link between the non-reaction aspect of mindfulness and meditation practice showed that VM was helpful for improving mindfulness and increasing life satisfaction among employees [24]. Singh et al. compared the mindfulness of the participants in their study before and after VM using the FFMQ and found that those who meditated were better able to stay calm, be aware of their thoughts without judgment, describe their feelings, and observe their experiences than those who did not meditate [25].

Overall, the evidence suggests that the practice of mindfulness meditation facilitates emotional balance, fear modulation, insight, sensory awareness, intuition, response flexibility, interpersonal attunement, empathy, and morality, helping individuals cope with situations in life [26]. By enhancing emotional regulation and, thus, creating mindful societies, meditation contributes to the UN’s Sustainable Development Goal 3 for positive health and well-being [27]. Furthermore, the data analyzed for the present study indicate that the change in awareness varied among the participants, but we observed a significant enhancement in mindfulness in both the employed group and the non-working group. We also observed significant changes in the non-working and retired participants, with a notable increase in those classified in the high mindfulness category following the VM intervention, from 0% to 83.3%. The unemployed participants demonstrated a significant increase in mindfulness, with 93.1% of them categorized as having strong mindfulness following the intervention. These findings are consistent with those of previous studies indicating that the practice of meditation, especially Vipassana, can significantly alter mindfulness posture, particularly among individuals who can dedicate significant time and have the flexibility to engage in intensive meditation retreats [28]. The analysis of the employed participants revealed a less pronounced rise in mindfulness after the VM retreat, but the difference was significant. The mean for these individuals increased from 59.73 before the retreat to 80.45 after, indicating a transition from a medium to a high level of mindfulness. Additionally, the strong results seen among full-time workers, who likely had limited time for long meditation sessions, show that VM can easily fit into many jobs as a way to practice mindfulness. Furthermore, the largest percentage of participants shifting from low to high mindfulness was in the student’s group, with those rating high on the mindfulness scale increasing from 9.1% before the VM retreat to 88.6% afterward. The increase in mindfulness among the student participants, who often faced considerable academic pressure, supports the idea that mindfulness practices such as Vipassana can help manage stress and improve focus, which, in turn, contribute to academic achievement and emotional and social health. Using the Wilcoxon signed-rank test for the scores before and after the VM session, we found strong evidence that this type of meditation improves awareness. The participants in all of the groups, irrespective of how mindful they were at the start of the study, showed much better results after the test, and the p-values (<0.001) indicated that these changes were unlikely to be attributable to chance. Furthermore, the change in mindfulness correlated with enhancement in both the awareness and the acceptance subscales of the PHLMS. The practice of being present, non-judgmental, and accepting of experiences, the core tenets of VM, thus appears to be a significant component of mindfulness that can positively influence psychological well-being, including emotional regulation, stress reduction, and overall well-being [13]. Mindfulness-based stress reduction programs in India and elsewhere, usually lasting for eight weeks and combining mindfulness meditation with gentle yoga, teach participants to react non-judgmentally to stressful events by focusing on automatic and dynamic stimuli (breath, body, eating, and walking) [28]. As individuals cultivate these skills, their top-down control processes regulate affective appraisals and reduce their stress responses [16]. 

Limitations of the article include lack of a control group, self-selection bias, and social desirability in self-reports. Future directions include similar studies with follow-ups of one, two, or six months, may be after a year as well as multicentric design would be better to evaluate the long-term effect of VM on individual and consistency by the participants. Better study designs such as randomized controlled trials can be used to evaluate the causal inference.

## Conclusions

The findings presented here indicate that VM markedly improves mindfulness, as assessed by the PHLMS, by increasing awareness and acceptance. These notable improvements among the groups of participants, students, employed and unemployed individuals, and retirees, demonstrate that VM effectively cultivated present-focused awareness and non-judgmental acceptance. These findings contribute to the expanding corpus of research endorsing mindfulness-based therapies for enhancing psychological well-being. The study demonstrated that VM markedly improved mindfulness in the participants irrespective of their backgrounds and, therefore, has substantial potential for developing acceptance and awareness.
